# Suppression of intrahepatic cholangiocarcinoma cell growth by SKI via upregulation of the CDK inhibitor p21

**DOI:** 10.1002/2211-5463.13489

**Published:** 2022-09-26

**Authors:** Etsushi Kawamura, Tsutomu Matsubara, Atsuko Daikoku, Sanae Deguchi, Masahiko Kinoshita, Hideto Yuasa, Hayato Urushima, Naoshi Odagiri, Hiroyuki Motoyama, Kohei Kotani, Ritsuzo Kozuka, Atsushi Hagihara, Hideki Fujii, Sawako Uchida‐Kobayashi, Shogo Tanaka, Shigekazu Takemura, Keiko Iwaisako, Masaru Enomoto, Y. H. Taguchi, Akihiro Tamori, Shoji Kubo, Kazuo Ikeda, Norifumi Kawada

**Affiliations:** ^1^ Department of Hepatology, Graduate School of Medicine Osaka Metropolitan University Japan; ^2^ Department of Anatomy and Regenerative Biology, Graduate School of Medicine Osaka Metropolitan University Japan; ^3^ Department of Hepato‐Biliary‐Pancreatic Surgery, Graduate School of Medicine Osaka Metropolitan University Japan; ^4^ Department of Medical Life Systems Doshisha University Graduate School of Life and Medical Sciences Kyoto Japan; ^5^ Department of Physics Chuo University Tokyo Japan

**Keywords:** cell cycle, cholangiocarcinoma, G1/S transition, p21, SKI

## Abstract

Cholangiocarcinoma (CC) has a poor prognosis and different driver genes depending on the site of onset. Intrahepatic CC is the second‐most common liver cancer after hepatocellular carcinoma, and novel therapeutic targets are urgently needed. The present study was conducted to identify novel therapeutic targets by exploring differentially regulated genes in human CC. MicroRNA (miRNA) and mRNA microarrays were performed using tissue and serum samples obtained from 24 surgically resected hepatobiliary tumor cases, including 10 CC cases. We conducted principal component analysis to identify differentially expressed miRNA, leading to the identification of miRNA‐3648 as a differentially expressed miRNA. We used an *in silico* screening approach to identify its target mRNA, the tumor suppressor Sloan Kettering Institute (*SKI*). SKI protein expression was decreased in human CC cells overexpressing miRNA‐3648, endogenous SKI protein expression was decreased in human CC tumor tissues, and endogenous *SKI* mRNA expression was suppressed in human CC cells characterized by rapid growth. SKI‐overexpressing OZ cells (human intrahepatic CC cells) showed upregulation of cyclin‐dependent kinase inhibitor p21 mRNA and protein expression and suppressed cell proliferation. Nuclear expression of CDT1 (chromatin licensing and DNA replication factor 1), which is required for the G1/S transition, was suppressed in SKI‐overexpressing OZ cells. SKI knockdown resulted in the opposite effects. Transgenic p21‐luciferase was activated in SKI‐overexpressing OZ cells. These data indicate SKI involvement in p21 transcription and that SKI–p21 signaling causes cell cycle arrest in G1, suppressing intrahepatic CC cell growth. Therefore, SKI may be a potential therapeutic target for intrahepatic CC.

## Graphical abstract

We analyzed clinical intrahepatic cholangiocarcinoma (CC) samples, focusing on the microRNA‐3648 and its target mRNA, the tumor suppressor Sloan Kettering Institute (SKI). We found that SKI was involved in the transcription of the cyclin‐dependent kinase inhibitor p21. SKI–p21 signaling may act as a brake, preventing cell cycle progression in G1 phase, resulting in the suppression of CC cell growth.

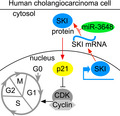

**Abbreviations**
CC: cholangiocarcinoma
CDK: cyclin‐dependent kinase
CDT1: chromatin licensing and DNA replication factor 1
CIP/KIP: CDK‐interacting protein/kinase inhibitory protein
DUSP: dual‐specificity phosphatase
ERK: extracellular signal‐regulated kinase
HCC: hepatocellular carcinoma
INK4: inhibitors of CDK4
MAPK: mitogen‐activated protein kinase
MEK: MAPK/ERK kinase
miRNA: microRNA
SKI: Sloan Kettering Institute
SMAD: smooth muscle actin plus mothers against decapentaplegic

Globally, liver cancer ranks second among all cancers when ranked by absolute years of life lost [1]. Intrahepatic cholangiocarcinoma (CC) accounts for 10–15% of liver cancers and represents the second‐most common liver cancer type after hepatocellular carcinoma (HCC) [2]. CC is associated with poor prognosis (≤ 20% 5‐year survival rate after resection) [3, 4]. Moreover, distant metastasis is commonly detected at the time of diagnosis, making patients ineligible for resection. Since 2010, systemic chemotherapy using gemcitabine plus cisplatin has been the international consensus first‐line treatment for unresectable CC [5]. Patients with CC that worsens after treatment with this regimen have limited treatment options. Genome sequencing revealed that CC driver genes differ depending on the site of onset (intra‐ or extrahepatic bile duct) [6]. Precision medicines, such as the fibroblast growth factor receptor 1–3 inhibitor pemigatinib, have been approved for use in Japan in patients with driver gene mutations; however, only 9–13% of patients with CC are eligible for targeted treatment [7, 8]. Although new methods for CC suppression are currently under investigation, such as sulfatase 2 monoclonal antibody [9], the discovery of additional therapeutic targets remains urgently necessary to treat patients with CC across all onset sites. Our research focuses on intrahepatic CC.

Active anticancer factors that regulate the cell cycle are being studied in various carcinomas. Cyclin‐dependent kinase (CDK) inhibitors bind to and inhibit the activity of the cyclin–CDK complex and include the inhibitors of CDK4 (INK4A–D) and the CDK‐interacting protein/kinase inhibitory protein (CIP/KIP) family (p21^Cip1^, p27^Kip1^, and p57^Kip2^). Among these, p21^Cip1^ (p21) inhibits the cell cycle at both G1 and G2 checkpoints, allowing time for DNA damage repair [10]. In human CC cells in which tumor enhancers (retinoid X receptor α and cyclooxygenase‐2) have been knocked down, p21 protein expression is upregulated, suppressing CC cell proliferation [11, 12]. This finding indicates that upregulation of *p21* gene expression may be the key to developing new CC‐specific therapies.

MicroRNAs (miRNAs) refer to a type of small noncoding RNA and are found in various specimens collected from patients with cancer, including tumor tissue, blood, and urine. miRNAs are involved in regulating the expression of various oncogenes associated with specific cancer types. In this study, we conducted an *in silico* screen to detect miRNAs in resected intrahepatic CC specimens and identify the mRNA sequences targeted by miRNAs of interest. In addition, we conducted functional analyses of identified tumor suppressor genes to examine whether they might serve as potential therapeutic targets. We focused on the mRNA encoding the tumor suppressor Sloan Kettering Institute (SKI), which was identified in our screen as being targeted by miRNA‐3648.

Transcription cofactors, such as SKI and SnoN, localize to the nuclei of most cancer cells. These molecules are widely expressed in *Drosophila* and humans [13] and exhibit significant activity during embryonic development, particularly during the growth and development of the central nervous system, extremities, and skeletal muscle [14]. However, the role of SKI in CC growth remains unclear. *SKI* was not included among the group of driver genes reported by the world's largest cohort study of patients with CC [15], and no studies in CC appear to have focused on *SKI*. In this study, we evaluated the relationship between SKI and p21 in human CC cells and examined whether SKI could serve as a new target for the treatment of intrahepatic CC.

## Materials and methods

### Patients and cell lines

A total of 10 intrahepatic CC tissue samples and 14 control samples were obtained by surgical resection at the Osaka Metropolitan University Hospital from July 2007 to June 2015 (Table 1). All samples were analyzed using miRNA and mRNA microarrays. All patients were diagnosed using tumor marker protein expression levels in serum (carcinoembryonic antigen, carbohydrate antigen 19‐9, α‐fetoprotein, and des‐γ‐carboxy prothrombin), computed tomography, and pathological examination by two independent pathologists. All patients provided written informed consent, and the Ethical Committee of Osaka Metropolitan University Graduate School of Medicine approved all aspects of this study in accordance with the 2008 revisions to the World Medical Association Declaration of Helsinki (Institutional Review Board number 3377). The human CC cell lines OZ and KKU100 were maintained in low‐glucose Dulbecco's modified Eagle medium (DMEM; Fujifilm Wako Pure Chemical Corporation, Osaka, Japan), supplemented with 10% fetal bovine serum (ThermoFisher Scientific, Waltham, MA, USA) and 1% penicillin–streptomycin (Fujifilm Wako Pure Chemical Corporation). All cell lines were maintained at 37 °C in a humidified atmosphere containing 5% CO_2_. The human CC cell lines were derived from two adenocarcinomas with pathological differences. The differentiated OZ (#1032) cell line was established from an intrahepatic CC case without hepatitis B virus/hepatitis C virus infection in Japan. The poorly differentiated KKU100 (#1568) cell line was established from a hilar CC case associated with Opisthorchiasis (liver fluke) infection by Khon Kaen University in Thailand (Japanese Collection of Research Bioresources Cell Bank, Osaka, Japan).

**Table 1:** Clinical details of patients with hepatobiliary tumor. HBV, hepatitis B virus; NA, not available.

Variables	No. of patients
Cholangiocarcinoma/hepatocellular carcinoma/benign^a^ (mean age, years)	10 (57.6^b^)/10 (66.8)/4 (74.3)
Female/male	7/17
Etiology^c^, HBV/HCV/diabetes/alcohol/NA	0/0/8/3/13
Alanine aminotransferase^d^ (IU·L^−1^), < 30/≥ 30	17/7
γ‐Glutamyltransferase^d^ (IU·L^−1^), < 80/≥ 80	14/10
Carcinoembryonic antigen^d^ (ng·mL^−1^), < 5/≥ 5/NA	18/3/3
Carbohydrate antigen 19–9^d^ (IU·mL^−1^), < 37/≥ 37/NA	14/7/3
α‐Fetoprotein^d^ (ng·mL^−1^), < 20/≥ 20	17/7
Des‐γ‐carboxy prothrombin^d^ (mAU·mL^−1^), < 40/≥ 40	14/10
Histological diagnosis (well/moderate to poor/NA); cholangiocarcinoma/hepatocellular carcinoma/benign^a^	10 (2/3/5)/10 (2/6/2)/4

^a^ Inflammatory pseudotumor, focal nodular hyperplasia of liver, angiomyolipoma of liver, adenomyomatous hyperplasia of gallbladder.

^b^ Includes four subjects younger than 50 years old.

^c^ All negative for hepatitis B surface antigen (HBsAg), anti‐hepatitis C virus (HCV) and chronic cholangitis.

^d^ Serological test within 1 month before surgery.

### miRNA and mRNA microarrays and miRNA selection

To select RNAs that are differentially regulated in tumor tissues, we performed miRNA and mRNA microarrays in resected tumor (T) and nontumor tissue (NT) samples obtained from patients with CC, particularly young individuals diagnosed with CC (YCC), patients diagnosed with HCC, and patients diagnosed with benign hepatobiliary tumors (Be) (Table S1 and Fig. S1).

Total RNA from tissue samples was extracted and purified using an miRNeasy mini kit (Qiagen, Hilden, Germany) for miRNA or an RNeasy mini kit (Qiagen) for mRNA, according to the manufacturer's instructions. Total RNA from serum samples was extracted and purified using an miRNeasy mini kit (Qiagen) for miRNA, after isolation by ExoQuick (System Biosciences, Palo Alto, CA, USA) for exosomes. mRNA labeling and hybridization were performed using the Agilent One‐Color Microarray‐Based Gene Expression Analysis protocol v6.5. Hybridization solution was added from the Agilent SurePrint G3 Human Gene Expression v3 8 × 60K Microarray Kit to detect 26 083 Entrez Genes (Agilent Technologies, Santa Clara, CA, USA) [16]. Comprehensive miRNA expression analysis was performed using a 3D‐Gene miRNA labeling kit and a Human miRNA Oligo Chip (Toray, Tokyo, Japan), which was designed to detect 2555 miRNAs registered in miRBase release 20 [17].

The fluorescence values “635 nm (IntegratedResult)” for miRNA and “gProcessedSignal (FE Raw files)” for mRNA were extracted from the microarray data and run in batches, from standardization to principal component analysis, using the code CCA/sample.R at main · tagtag/CCA, last committed on 17 June 2022. We used the “prcomp” function of R [18] and the first and second principal components (PC1 and PC2, respectively) to understand the microarray results. We performed dimensionality reduction to better observe differences in gene expression in each group and expanded expression patterns into two dimensions using PC2 for both miRNA and mRNA. In the plots of tumors obtained from the four types of patients (CC‐T, YCC‐T, HCC‐T, and Be‐T in Fig. S2A), differences were observed in the distances for both miRNA and mRNA, suggesting that the gene expression levels could discriminate between these groups.

We used the code to expand the miRNA (Fig. S2B) and mRNA (Fig. S2C) expression patterns into two dimensions based on PC1 and PC2, resulting in the extraction of 24 miRNAs (Table S2 and Fig. S3) [19, 20, 21, 22, 23, 24, 25, 26, 27, 28] and 141 mRNAs (Table S3). We also used identified target mRNAs for extracted miRNAs, with a particular focus on mRNAs associated with digestive cancer, resulting in the identification of five mRNA candidates for inclusion in the *in vitro* analysis. We identified cancer‐related pathways (Tables [Link], [Link]) using the mirPath database (https://dianalab.e‐ce.uth.gr/html/mirpathv3/index.php?r=mirpath, last accessed on 26 June 2022) for miRNA and Integrated Molecular Pathway Level Analysis database (http://impala.molgen.mpg.de, last accessed on 10 July 2022) for mRNA. We used the following mathematical method to select differentially expressed miRNAs and mRNAs, using a “semi‐supervised learning” approach in serum exosomal miRNA samples that are not used as labels [29]:

*x*_*ij*_ and *x*_*kj*_ represent the *i*th mRNA expression and the *k*th miRNA expression of the *k*th patient, respectively. These factors were normalized as $\sum_{i=1}^{N}x_{\mathrm{ij}}=\sum_{k=1}^{K}x_{\mathrm{kj}}=0,\sum_{i=1}^{N}x_{\mathrm{ij}}^{2}=N,\;\text{and}\sum_{k=1}^{K}x_{\mathrm{kj}}^{2}=K,$ where *N* and *K* are the total numbers of mRNAs and miRNAs, respectively. The *l*th principal component, attributed to *i*th mRNA, $u_{\mathrm{li}}^{\text{mRNA}}$, and the *k*th miRNA, $u_{\mathrm{lk}}^{\text{miRNA}}$, were the *i*th and *k*th components, respectively, of the *l*th eigenvector of the gram matrices $\sum_{j=1}^{M}x_{\mathrm{ij}}x_{i^{\prime }j}$ and $\sum_{j=1}^{M}x_{\mathrm{kj}}x_{k^{\prime }j}$, expressed as

$$\sum_{i=1}^{N}u_{\mathrm{lj}}^{\text{mRNA}}\sum_{j=1}^{M}x_{\mathrm{ij}}x_{ij^{\prime }}=\lambda_{l}^{\text{mRNA}}u_{li^{\prime }}^{\text{mRNA}},$$

$$\sum_{k=1}^{K}u_{\mathrm{kj}}^{\text{miRNA}}\sum_{j=1}^{M}x_{\mathrm{kj}}x_{k^{\prime }j}={\lambda_{l}^{\text{miRNA}}u}_{lj^{\prime }}^{\text{miRNA}},$$

where *M* is the total number of patients. The *l*th principal component loading attributed to the *j*th sample using mRNA, $v_{\mathrm{lj}}^{\text{mRNA}}$, and miRNA, $v_{\mathrm{lj}}^{\text{miRNA}}$, was the *j*th component of the *l*th eigenvector of the covariant matrices $\sum_{i=1}^{N}x_{\mathrm{ij}}x_{ij^{\prime }}$ and $\sum_{k=1}^{K}x_{\mathrm{kj}}x_{kj^{\prime }}$ expressed as

$$\sum_{j=1}^{M}v_{\mathrm{lj}}^{\text{mRNA}}\sum_{i=1}^{N}x_{\mathrm{ij}}x_{ij^{\prime }}=\lambda_{l}^{\text{mRNA}}v_{lj^{\prime }}^{\text{mRNA}},$$

$$\sum_{j=1}^{M}v_{\mathrm{lj}}^{\text{miRNA}}\sum_{k=1}^{K}x_{\mathrm{kj}}x_{kj^{\prime }}={\lambda_{l}^{\text{miRNA}}v}_{lj^{\prime }}^{\text{miRNA}}.$$

To select mRNAs and miRNAs that are negatively correlated, we identified a pair of $v_{lj^{\prime }}^{\text{mRNA}}$ and $v_{lj^{\prime }}^{\text{miRNA}}$ that were mutually correlated. We found that *l* = 2 satisfies this condition. Thus, PC2 was used for miRNA and mRNA selection. We attributed *P*‐values to the *k*th miRNA and the *i*th miRNA using

$$P_{k}=P_{\chi^{2}}\left[>{\left(\frac{u_{2k}}{\sigma_{2}}\right)}^{2}\right],$$

$$P_{i}=P_{\chi^{2}}\left[>{\left(\frac{u_{2i}}{\sigma_{2}}\right)}^{2}\right],$$

where $P_{\chi^{2}}\left[>x\right]$ is the cumulative χ^2^ distribution in which the argument is larger than *x*, and σ_2_ is the standard deviation. *P*_*k*_ and *P*_*i*_ were corrected using the Benjamini–Hochberg criterion. Associated miRNAs with an adjusted *P*_*k*_ < 0.01 and mRNAs with an adjusted *P*_*i*_ < 0.01 were selected.

### Cell transfection

The human CC cell lines OZ and KKU100 (2 × 10^5^ cells/well) were seeded in 12‐well plates 1 day before transfection. Transfections were performed in Opti‐MEM (ThermoFisher Scientific) using Lipofectamine 3000 (ThermoFisher Scientific) for the transfection of pCMV6‐FLAG‐tagged human SKI expression plasmids (SKI plasmid; OriGene, Rockville, MD, USA) and pCMV6‐empty and Lipofectamine RNAiMAX (ThermoFisher Scientific) for the transfection of human miRNA‐3648 mimic, the miRNA mimic negative control (Dharmacon, Lafayette, CO, USA), SKI siRNA (Abcam, Cambridge, UK), p21 siRNA (Dharmacon), and siCNT (ThermoFisher Scientific). All transfected cells were incubated at 37 °C according to the manufacturer's instructions. The amount of transfected plasmid or RNA used in each well was equalized by the addition of empty plasmid or siCNT, respectively.

### Quantitative real‐time polymerase chain reaction

RNA was extracted from cells using TRIzol reagent (ThermoFisher Scientific) and Direct‐zol RNA Miniprep (Zymo Research, Irvine, CA, USA). cDNA was generated from total RNA in a PCR Thermal Cycler Dice Gradient TP600 (TaKaRa Bio, Shiga, Japan) using Superscript III (ThermoFisher Scientific), dNTP Mixture (TaKaRa Bio), and random primers (ThermoFisher Scientific) for mRNA, whereas Mir‐X miRNA First‐Strand synthesis kit (TaKaRa Bio) was used for miRNA. Quantitative real‐time polymerase chain reaction was performed with the FAST SYBR Green PCR Master Mix (ThermoFisher Scientific) for mRNA or TB Green Advantage qPCR Premix for miRNA (TaKaRa Bio) using a 7500 Fast Real‐Time PCR System (ThermoFisher Scientific). The human primer sequences are shown in Table 2. Expression values were calculated using the comparative *C*_t_ method. mRNA or miRNA expression levels are presented as relative expression, following normalization against the expression levels of 18S rRNA or U6, respectively, which served as endogenous controls.

**Table 2:** Primers for quantitative real‐time polymerase chain reaction used in the present study. *p21*, *p21Waf* or *p21Cip1* or cyclin‐dependent kinase inhibitor 1A (*Cdkn1a*); *p27*, *p27Kip1* or *Cdkn1b*; *DUSP*, dual‐specificity phosphatase; 18S, 18S ribosomal RNA; microRNA‐3648, microRNA 3648‐1 or 2; U6, U6 small nuclear RNA.

Human gene	Forward (5′–3′)	Reverse (5′–3′)
*SKI*	CGACGTGAAGGAGAAATTCG	GTTTTGGGTCTTATGGAGGC
*p21*	AGTCAGTTCCTTGTGGAGCC	CATGGGTTCTGACGGACAT
*p27*	TAATTGGGGCTCCGGCTAACT	TGCAGGTCGCTTCCTTATTCC
*DUSP2*	GGGCTCCTGTCTACGACCA	GCAGGTCTGACGAGTGACTG
*DUSP6*	GAAATGGCGATCAGCAAGACG	CGACGACTCGTATAGCTCCTG
18S	CAGCCACCCGAGATTGAGCA	TAGTAGCGACGGGCGGTGTG
microRNA‐3648	AGCCGCGGGGATCGCCGAGGG	Not disclosed^a^
U6	Not disclosed^b^	Not disclosed^c^

^a^ mRQ 3′primer.

^b^ U6 forward primer.

^c^ U6 reverse primer (TaKaRa Bio).

### Western blotting

Cells were homogenized with radioimmunoprecipitation assay lysis buffer (50 mm Tris/HCl at pH 7.5, 150 mm NaCl, 1% Triton X‐100, 1% sodium dodecyl sulfate) containing the protease inhibitor cocktail cOmplete Mini (Roche, Basel, Switzerland) and phosphatase inhibitors (1 mm sodium fluoride, 1 mm β‐glycerol phosphate, and 1 mm sodium vanadate). Protein samples were separated by 8–15% sodium dodecyl sulfate/polyacrylamide gel electrophoresis and transferred to polyvinylidene difluoride membranes using standard western blotting techniques with a Powerpac basic mini electrophoresis system (Bio‐Rad Laboratories, Richmond, CA, USA). After blocking with 5% skim milk, the membranes were probed with primary antibodies diluted 1 : 1000 in 3% bovine serum albumin/Tris‐buffered saline/0.1% Tween‐20 (Merck, Darmstadt, Germany) (3% BSA/TBST) at 4 °C overnight, followed by 1‐h incubation at room temperature with horseradish peroxidase–conjugated anti‐rabbit (#7074) or anti‐mouse (#7076) secondary antibodies (Cell Signaling Technology, Lane Danvers, MA, USA), diluted 1 : 5000 in 3% BSA/TBST. Immunoreactive bands were visualized using the ImmunoStar Zeta or ImmunoStar LD peroxidase luminescent substrates (Fujifilm Wako Pure Chemical Corporation) and detected using the LAS4000 mini‐imaging system (GE Healthcare, Chicago, IL, USA). WB Stripping Solution (Nacalai Tesque, Kyoto, Japan) was used to remove the antibodies from the membrane. Protein expression was normalized against the expression of endogenous controls, either glyceraldehyde 3‐phosphate dehydrogenase (GAPDH) or β‐actin, and is presented as the relative expression level.

Primary antibodies were obtained as follows: anti‐SKI (#33693), anti‐dual‐specificity phosphatase (DUSP)2 (#32776), anti‐DUSP6 (#377070), anti‐lamin B1 (#374015), and antichromatin licensing and DNA replication factor 1 (CDT1; #365305) (Santa Cruz Biotechnology, Santa Cruz, CA, USA); antiphosphorylated‐smooth muscle actin plus mothers against decapentaplegic (SMAD)3 (#9520), anti‐SMAD3 (#9513), antiphosphorylated mitogen‐activated protein kinase (MAPK)/extracellular signal‐regulated kinase (ERK) kinase (MEK) 1/2 (#9154), anti‐MEK1/2 (#8727), antiphosphorylated‐ERK1/2 (#4370), anti‐ERK1/2 (#4695) anti‐p21 (#2947) and anti‐p27 (#3686) (Cell Signaling Technology); anti‐β‐actin (#A2228), and anti‐GAPDH (#374) (Merck).

### Immunohistochemistry and immunofluorescence analyses

For immunohistochemistry, 5‐μm‐thick sections obtained from paraffin‐embedded blocks of human CC tissue samples were deparaffinized and treated with 1% H_2_O_2_‐MeOH for 1 h. After blocking with 3% BSA/TBST, sections were incubated with a primary antibody against SKI (#HPA066567; Atlas Antibodies, Bromma, Sweden; 1 : 100 in 3% BSA/TBST) at 4 °C overnight, followed by incubation with the secondary antibody Histofine Simple Stain Mouse MAX‐PO (Nichirei Biosciences, Tokyo, Japan) for 1 h at room temperature. The signal was visualized with 3,3′‐diaminobenzidine. The mean intensity of the SKI signal was quantified using imagej 1.52k (National Institutes of Health, Bethesda, MD, USA). The SKI expression level was calculated per unit of tumorous or nontumorous area in the bile duct as detected by immunostaining.

For immunofluorescence analysis, OZ cells (5 × 10^4^ cells/well) were seeded in a 6‐well plate 1 day before transfection. Cells were transfected with 340 ng·mL^−1^ of SKI plasmid using Lipofectamine 3000 or 10 nm of SKI siRNA using Lipofectamine RNAiMAX. After 48 h of transfection, the cells were fixed with 4% paraformaldehyde, and the wells were blocked with 3% BSA/TBST. The cells were incubated with a primary antibody against CDT1 (#14382; Proteintech, Rosemont, IL, USA; 1:100 in 3% BSA/TBST) at 4 °C overnight, followed by incubation with Alexa Fluor 594 goat anti‐rabbit secondary antibody (#A‐11037; ThermoFisher Scientific; 1:400 in 3% BSA/TBST) for 1 h at room temperature. Nuclei were visualized with 4′,6‐diamidino‐2‐phenylindole (#D523; Dojindo, Kumamoto, Japan).

The cells were photographed using a BZ‐X710 microscope for immunohistochemistry and a BX53F microscope for immunofluorescence (both Olympus, Tokyo, Japan).

### Cell proliferation assay

OZ or KKU100 cells (2 × 10^5^ cells/well) were seeded in a 12‐well plate 1 day before transfection. Cells were transfected with SKI plasmid (170, 340, and 500 ng·mL^−1^) using Lipofectamine 3000 or SKI siRNA (3.3 and 10 nm) using Lipofectamine RNAiMAX. After 24 h of transfection, (Day 0) OZ or KKU100 cells were trypsinized and seeded (5 × 10^3^ cells/well) in a 96‐well plate in 100 μL RPMI 1640 culture medium (ThermoFisher Scientific). After 24 h of seeding, the reagent solution for 3‐(4,5‐dimethylthiazol‐2‐yl)‐2,5‐diphenyltetrazolium bromide (MTT) assay (5 mg·mL^−1^; Biotium, Fremont, CA, USA) was added to each well, and dimethyl sulfoxide (100 μL) was added to sonically dissolve the resulting formazan. The cells were incubated for 3 h at 37 °C. The CCK‐8 assay was performed using the same timing relative to transfection and seeding described for the MTT assay. The CCK‐8 reagent solution (10 μL; Dojindo) was added to each well, and the cells were incubated for 30 min at 37 °C. After incubations above, absorbance was measured at 570 nm for MTT assays and at 450 nm for CCK‐8 assays using a Varioskan LUX microplate reader (ThermoFisher Scientific).

### Transwell assay

KKU100 or OZ cells (2 × 10^5^ cells/well) were seeded in a 12‐well plate 1 day before transfection. Cells were transfected with 500 ng·mL^−1^ of SKI plasmid using Lipofectamine 3000 (ThermoFisher Scientific). After 24 h of transfection, OZ or KKU100 cells were trypsinized and seeded (3 × 10^4^ cells/well) in the upper compartment of Falcon inserts containing an 8‐μm‐pore polyethylene terephthalate membrane (#353182; Corning, Corning, NY, USA) in a 12‐well plate. Culture medium containing 10% fetal bovine serum was added to the lower compartment. After 24 h, the cells remaining on the upper surface were rinsed twice with phosphate‐buffered saline. Cells that had migrated across the membrane were fixed and stained using 0.05% crystal violet methanol and photographed with a BZ‐X710 microscope (Olympus).

### Reporter gene assay

The p21 gene promoter region from −2475 nt to +93 nt was amplified in the bacterial artificial chromosome (BAC) clone RP11‐845C9 (Advanced GenoTechs, Ibaraki, Japan) using PrimeSTAR Max DNA Polymerase (TaKaRa Bio) with forward primer 5′‐AAGGTACCGAGCCTTCCTCACATCCTCCTTCTTCAG‐3′ and reverse primer 5′‐AGAAGCTTTCTCTCACCTCCTCTGAGTGCCTCGGTG‐3′. A p21 gene reporter plasmid was generated by inserting the amplicon into the *Kpn*I/*Hind*III site of a firefly luciferase reporter pGL4.10 vector (Promega, Madison, WI, USA). OZ cells (5 × 10^4^ cells/well) were seeded in 24‐well plates 1 day before transfection. Cells were transfected with 200 ng·mL^−1^ of SKI plasmid, together with 30 ng·mL^−1^ p21 gene reporter plasmid and 10 ng·mL^−1^ pRL‐SV40 *Renilla* luciferase control reporter vector (Promega), using Lipofectamine 3000 (ThermoFisher Scientific). After 48 h of transfection, firefly and *Renilla* luciferase activities were measured with a Dual‐Luciferase Reporter Assay System (Promega). Luciferase activity was normalized for transfection efficiency using the corresponding *Renilla* luciferase activity.

### Cell cycle assay

To understand at which stage of the cell cycle SKI–p21 signaling affects CC growth, we performed flow cytometry on two complex phases, G0/G1 and S/G2/M. KKU100 or OZ cells (5 × 10^4^ cells/well) were seeded in a 6‐well plate 1 day before transfection. KKU100 cells were transfected with 500 ng·mL^−1^ of SKI plasmid using Lipofectamine 3000 (ThermoFisher Scientific), and OZ cells were transfected with 10 nm of SKI siRNA using Lipofectamine RNAiMAX (ThermoFisher Scientific). After 48–58 h of transfection, the cells were starved in low‐glucose DMEM (Fujifilm Wako Pure Chemical Corporation) supplemented with 0.5% fetal bovine serum (ThermoFisher Scientific), followed by trypsinization and centrifugation at 72‐h post‐transfection. The supernatant was discarded, and cells were suspended in phosphate‐buffered saline and incubated with fluorescent particle allophycocyanin and a cyanine dye (APC‐Cy7 A; Dojindo) for 15 min. Diploid and tetraploid fractions of SKI‐overexpressing KKU100 cells (Fig. S4A) or SKI‐knockdown OZ cells (Fig. S4B) were detected with an LSR II flow cytometer (BD Biosciences, Franklin Lakes, NJ, USA).

### Statistical analysis

Analysis results are presented as the fold change relative to the negative control, and differences between groups were analyzed by *t*‐test (for two groups) or analysis of variance (ANOVA) (for three groups). Statistical analysis was performed using prism version 8.01 (GraphPad Software, San Diego, CA, USA), and principal component analysis was performed using R (R Foundation for Statistical Computing, Vienna, Austria). *P* < 0.05 was considered significant.

## Results and Discussion

### Endogenous SKI mRNA and protein are expressed at low levels in human CC tissue and cells

**Fig. 1:**
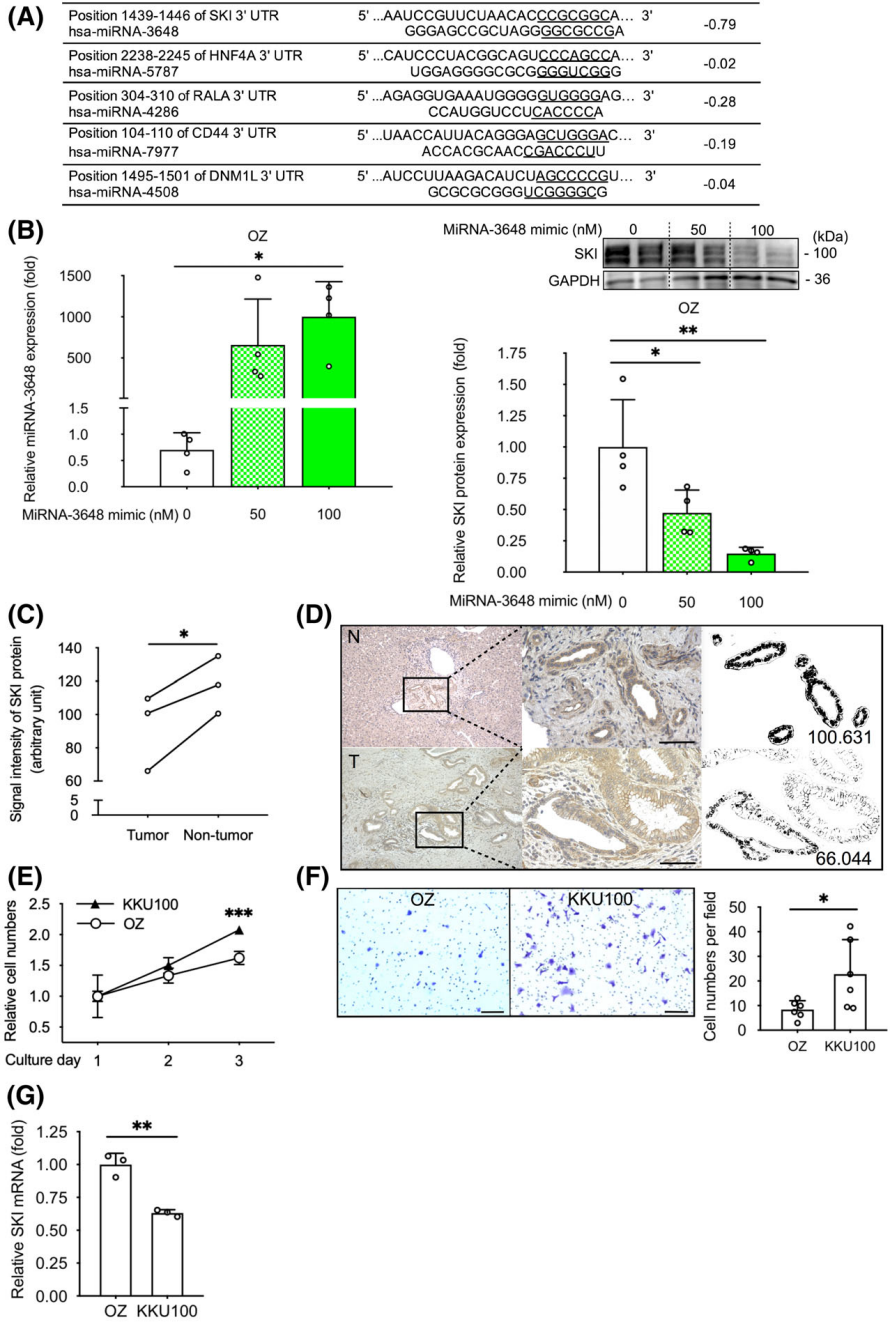
Endogenous *SKI* mRNA and protein are expressed at low levels in human cholangiocarcinoma tissue and cells. (A) The predicted pairing between target human 3′‐untranslated regions of mRNA sequences (top, underlined) and human microRNA (miRNA) sequences (bottom, underlined) and the cumulative weighted context++ scores (right column), which indicate target effectiveness according to *in silico* screening (targetscan.org). (B) Quantitative real‐time polymerase chain reaction analysis of miRNA‐3648 and western blots performed in miRNA‐3648‐overexpressing (50 and 100 nm miRNA mimic) human cholangiocarcinoma OZ cell and control cells. GAPDH served as a loading control (right panels). Endogenous functions were analyzed by multiple methods (C–G). (C) Immunohistochemical analysis of SKI in the bile duct epithelium of resected cholangiocarcinoma cases. Mean SKI signal intensity was calculated from tissue samples from three patients, including the case shown in (D). (D) The boxed area shows a higher magnification of the tissue sample. Upper row, nontumor area (N); lower row, tumor area (T); scale bars represent 100 μm using high‐powered magnification (×400); numbers represent the SKI signal intensity. (E) The number of human cholangiocarcinoma OZ and KKU100 cells on the indicated days of culture after seeding (*n* = 4, using MTT). (F) Transwell migration assay for OZ and KKU100 cells (*n* = 6); scale bars, 100 μm (left); and quantitation (right). (G) Quantitative real‐time polymerase chain reaction analysis showing the relative levels of *SKI* mRNA in OZ and KKU100 cells (*n* = 3). Symbols and bars represent the mean ± standard deviation (B, F, G) or mean ± standard error (E). All cells were transfected with equal amounts of total RNA through the addition of siCNT (B). *P < 0.05, **P < 0.01, ***P < 0.001; one‐way ANOVA with Dunnett's test (B), paired *t*‐test (C), or unpaired *t*‐test (E–G). *HNF4A*, hepatocyte nuclear factor 1 alpha; *RALA*, RAS‐like proto‐oncogene A; *CD44*, CD44 molecule (Indian blood group); *DNM1L*, dynamin 1‐like.

To explore the gene expression profile in human intrahepatic CC tissues, we conducted miRNA and mRNA microarrays, principal component analysis, and *in silico* screening. Principal component analysis indicated that 24 miRNAs might be differentially regulated in tumors compared with normal samples (Figs [Link], [Link]). Consequently, five miRNAs, miRNA‐3648, miRNA‐5787, miRNA‐4286, miRNA‐7977, and miRNA‐4508, and their potential mRNA targets, including *SKI*, hepatocyte nuclear factor 1 alpha, RAS‐like proto‐oncogene A, CD44 molecule (Indian blood group), and dynamin 1‐like (Fig. 1A), were selected according to previous reports showing relationships with digestive cancers other than CC. Of these five mRNAs, *SKI* mRNA showed the lowest cumulative weighted context++ score (−0.79), which indicates that miRNA‐3648 and *SKI* mRNA have the strongest effector:target relationship among the five miRNA–mRNA pairs [30]. Furthermore, in the CC patient‐derived OZ cell line, SKI protein expression decreased when miRNA‐3648 was overexpressed compared with control cells (6.7‐fold decrease induced by transfection with 100 nm miR mimic; Fig. 1B). Thus, miRNA‐3648 decreased SKI protein levels in CC cells, as predicted by the *in silico* screen.

To assess endogenous SKI expression, we examined surgically resected CC tissue and human CC cell lines defined as low‐malignancy (OZ) or high‐malignancy (KKU100). SKI signal intensity was detected by immunostaining in the tumor‐associated regions of surgically resected bile ducts from patients with CC, revealing attenuated expression compared with nontumorous, normal bile duct samples (1.3‐fold decrease, Fig. 1C). Figure 1D shows representative images from the case whose data are included in Fig. 1C. KKU100 cells displayed a 1.28‐fold increase in proliferation compared with OZ cells on culture day 3 (assessed by MTT assay, Fig. 1E). The cell migratory ability of KKU100 cells increased by 2.71‐fold that of OZ cells (Fig. 1F). The expression level of *SKI* mRNA in KKU100 cells was 1.58‐fold lower than that in OZ cells (Fig. 1G). These data indicate that OZ cells with higher endogenous SKI expression showed reduced proliferative and migratory abilities than KKU100 cells with lower endogenous SKI expression. The findings (Fig. 1C–G) that endogenous SKI protein and mRNA expression decreased in both tumorous tissue regions and in cells with rapid growth led us to predict that SKI may play a role in CC growth.

### SKI suppresses cell proliferation in human CC

**Fig. 2:**
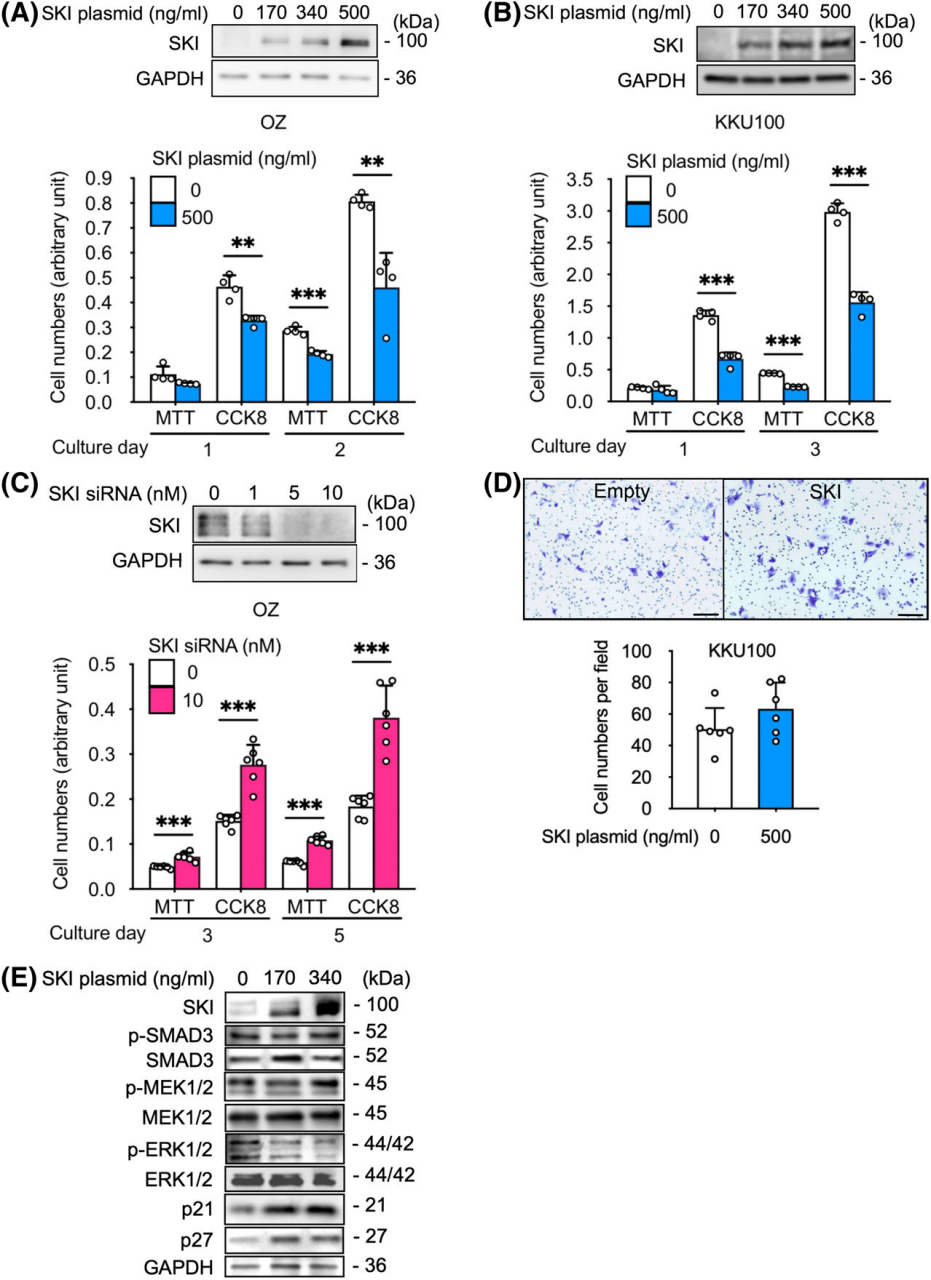
Sloan Kettering Institute suppresses cell proliferation and regulates key proliferation–related signaling proteins in human cholangiocarcinoma. SKI overexpression in (A) OZ and (B) KKU100 human cholangiocarcinoma cell lines were assessed by western blot analysis (top, 0–500 ng·mL^−1^ plasmid) and cell proliferation assays (bottom, MTT and CCK‐8, 500 ng·mL^−1^ plasmid; *n* = 4). (C) SKI knockdown in OZ cells was assessed by western blot analysis (top, 0–10 nm siRNA) and cell proliferation assays (bottom, MTT and CCK‐8, 10 nm siRNA, *n* = 6). (D) Transwell migration assay comparing SKI‐overexpressing (500 ng·mL^−1^ plasmid) KKU100 cells with control cells (*n* = 6); scale bar, 100 μm. (E) Qualitative western blot analysis of cell proliferation–related proteins, including phosphorylated SMAD3, total SMAD3, phosphorylated MEK1/2, total MEK1/2, phosphorylated ERK1/2, total ERK1/2, p21, and p27 in SKI‐overexpressing (170/340 ng·mL^−1^ plasmid) OZ cells and control cells. GAPDH (A–C, E) served as a loading control. Symbols and bars represent the mean ± standard deviation (A–D). All cells were transfected with equal amounts of total plasmid or RNA through the addition of empty plasmid or siCNT, respectively (A–E). **P < 0.01, ***P < 0.001; unpaired *t*‐test (A–D). CCK‐8, cell counting kit 8; MEK, MAPK/ERK kinase.

To verify the role of SKI, we evaluated the relationship between SKI overexpression (through plasmid transfection) and CC cell growth (by assessing proliferation and migration). The proliferation analysis demonstrated that SKI‐overexpressing CC cell lines showed inhibited cell growth compared with control cells (1.52‐fold decrease assessed by MTT assay and 1.75‐fold decrease assessed by CCK‐8 assay on day 2 in OZ cells; Fig. 2A; 1.93‐fold decrease assessed by MTT assay, and 1.91‐fold decrease assessed by CCK‐8 assay on day 3 in KKU100 cells; Fig. 2B). Cell growth was promoted in SKI‐knockdown OZ cells compared with control cells (1.82‐fold increase assessed by MTT assay and 2.11‐fold increase assessed by CCK‐8 assay on day 5; Fig. 2C). By contrast, the migration of SKI‐overexpressing KKU100 cells was not inhibited compared with control cells (Fig. 2D).

These data show that the proliferation of human CC cell lines was inhibited by SKI overexpression and promoted by SKI knockdown, whereas cell migration was not inhibited by SKI overexpression in KKU100 cells, which have a higher migratory ability than OZ cells. Therefore, we hypothesized that SKI might be involved in MAP‐ERK signaling or TGF‐β/SMAD signaling or interact with the CDK inhibitors that regulate cell proliferation; however, SKI is unlikely to be involved with the signaling pathways that control cell migration [31].

The OZ cell line is better balanced than the KKU100 cell line in two ways: The OZ cell line was derived from a typical intrahepatic CC case identified in a Japanese man, and the OZ cell line showed the more efficient transfection of SKI plasmids than the KKU100 cell line. Therefore, we performed all subsequent experiments using the OZ cell line.

### SKI is involved in the regulation of key cell proliferation–related signaling proteins in human CC

To examine the relationship between SKI and key regulatory signaling factors involved in cell proliferation, we qualitatively assessed the protein expression of TGF‐β/SMAD, MAPK/ERK, and cell cycle signaling factors in OZ cells. SKI has been reported to suppress TGF‐β/SMAD signaling in other carcinomas [32, 33]. However, Fig. 2E indicates that the protein expression levels of phosphorylated and total SMAD3 were not suppressed in SKI‐overexpressing OZ cells. By contrast, SKI‐overexpressing OZ cells suppressed the protein expression of phosphorylated ERK1/2 (MAP‐ERK signaling) and upregulated the protein expression of p21 and p27 (CDK inhibitors).

### SKI promotes p21 expression in human CC cells

**Fig. 3:**
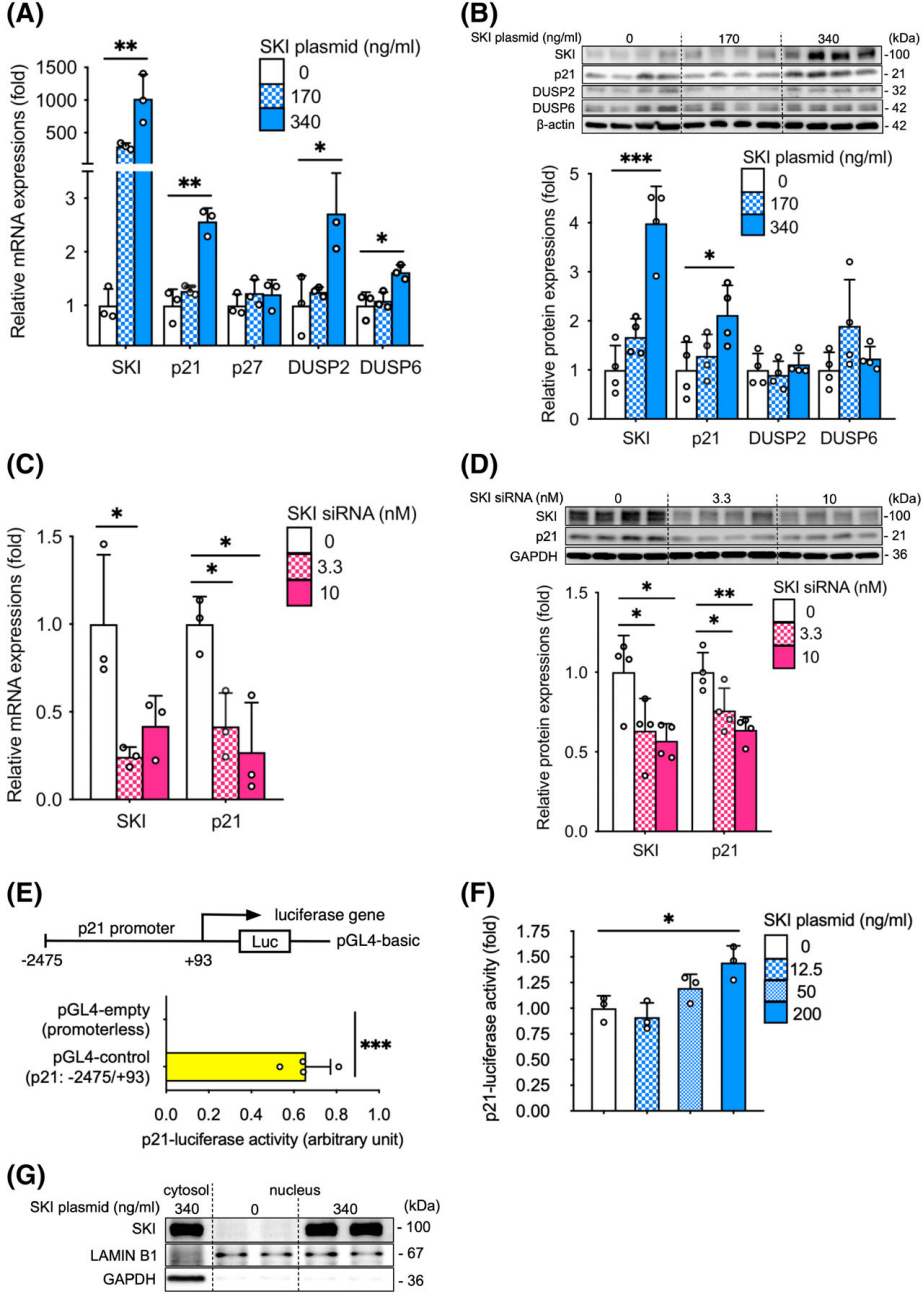
Sloan Kettering Institute promotes p21 expression and activates the *p21* promoter in human cholangiocarcinoma cells. (A) Quantitative analysis of *p21*, *p27*, dual‐specificity phosphatase (*DUSP*)*2*, and *DUSP6* mRNA (*n* = 3) and (B) p21, DUSP2, and DUSP6 protein (*n* = 4) in SKI‐overexpressing (0, 170, and 340 ng·mL^−1^ plasmid) human cholangiocarcinoma OZ cells. (C) Quantitative analysis of p21 mRNA (*n* = 3) and (D) protein (*n* = 4) in SKI‐knockdown (0, 3.3, and 10 nm siRNA) OZ cells. (E) Diagram showing the luciferase reporter gene containing the transcription start sequence of the *p21* promoter and luciferase activity in OZ cells (*n* = 4). (F) Luciferase activity in human cholangiocarcinoma OZ cells co‐transfected with 30 ng·mL^−1^ of firefly luciferase reporter pGL4‐p21 plasmid, 200 ng·mL^−1^ of SKI plasmid, and 10 ng·mL^−1^ of pRL‐SV40 *Renilla* luciferase control reporter vector (*n* = 3). (G) Western blot analysis showing the localization of SKI in the nuclear fraction of SKI‐overexpressing (0 or 340 ng·mL^−1^ plasmid) OZ cells. β‐actin (B), GAPDH (D, G), or lamin B1 (G) served as loading controls. Symbols and bars represent the mean ± standard deviation (A–F). All cells were transfected with equal amounts of total plasmid or RNA through the addition of empty plasmid or siCNT, respectively (A–D, F, G). *P < 0.05, **P < 0.01, ***P < 0.001; one‐way ANOVA with Dunnett's test (A–D, and F) or unpaired *t*‐test (E).

To verify the relationships between SKI overexpression and ERK1/2 dephosphorylation and between SKI overexpression and CDK inhibitor (p21 and p27) upregulation, we quantitatively analyzed mRNA and protein expression levels in OZ cells transfected with the SKI plasmid. p21 mRNA (2.57‐fold) and protein (2.12‐fold) increased in SKI‐overexpressing (340 ng·mL^−1^ plasmid) OZ cells compared with control cells (Fig. 3A,B); the opposite effects were observed following SKI knockdown (10 nm siRNA) in OZ cells (3.7‐fold decrease for *p21* mRNA and 1.56‐fold decrease for p21 protein; Fig. 3C,D). However, *p27* mRNA did not increase in SKI‐overexpressing OZ cells compared with control cells (Fig. 3A). *DUSP2* and *DUSP6* encode ERK1/2‐specific phosphatases, and the mRNA expression levels of these two factors increased in SKI‐overexpressing OZ cells compared with control cells (2.72‐fold and 1.62‐fold, respectively), but no increase in protein expression was observed (Fig. 3A,B). The DUSP proteins regulate the inactivation of the best‐known MAPKs, Jun N‐terminal kinase, p38, and ERK. We analyzed whether SKI overexpression promotes the expression of six *DUSP* mRNAs, *DUSP2*, *DUSP4*, *DUSP5*, *DUSP6*, *DUSP7*, and *DUSP9*, which are strongly involved in ERK dephosphorylation; however, only *DUSP2* and *DUSP6* showed significant differences (data not shown except for *DUSP2* and *DUSP6*) [34]. Because these analyses indicated that SKI overexpression affected both the mRNA and protein levels of p21, in contrast with the other examined factor, we focused our attention on the role of SKI in the regulation of p21 expression.

### SKI activates the p21 promoter in human CC cells

To verify the relationship between SKI and p21, we generated a plasmid vector (pGL4‐p21) containing the predicted transcription start site of the *p21* promoter (spanning −2.5 to +0.09 kbp of the promotor region; Fig. 3E). pGL4‐p21 (30 ng·mL^−1^) and SKI plasmids (200 ng·mL^−1^) were co‐transfected into OZ cells, and luciferase activity was measured. p21 luciferase activity was enhanced in SKI‐overexpressing OZ cells compared with control cells (1.5‐fold increase; Fig. 3F). Western blotting showed that SKI expression was enhanced in the nuclear fraction of SKI‐overexpressing OZ cells, indicating that SKI protein functions in the nucleus of CC cells (Fig. 3G). Taken together, these data indicate that SKI activates the *p21* promoter and is involved in the transcription of *p21* in the nucleus of CC cells.

SKI functions have been reported in cell types other than CC, including SKI‐like protein expression independent of p53 (embryonic stem cells [35]), tumor growth inhibition via Hippo signaling (breast cancer [36]), and tumor growth promotion via suppression of TGF‐β expression (esophageal squamous cell carcinoma [32] and malignant melanoma [33]). Our data (Figs 2 and 3) indicate the possibility that SKI inhibits tumor growth in human CC cells through the promotion of CDK inhibitor p21 expression.

### SKI suppresses cell proliferation via p21 upregulation and inhibits nuclear distribution of CDT1 in human CC cells

**Fig. 4:**
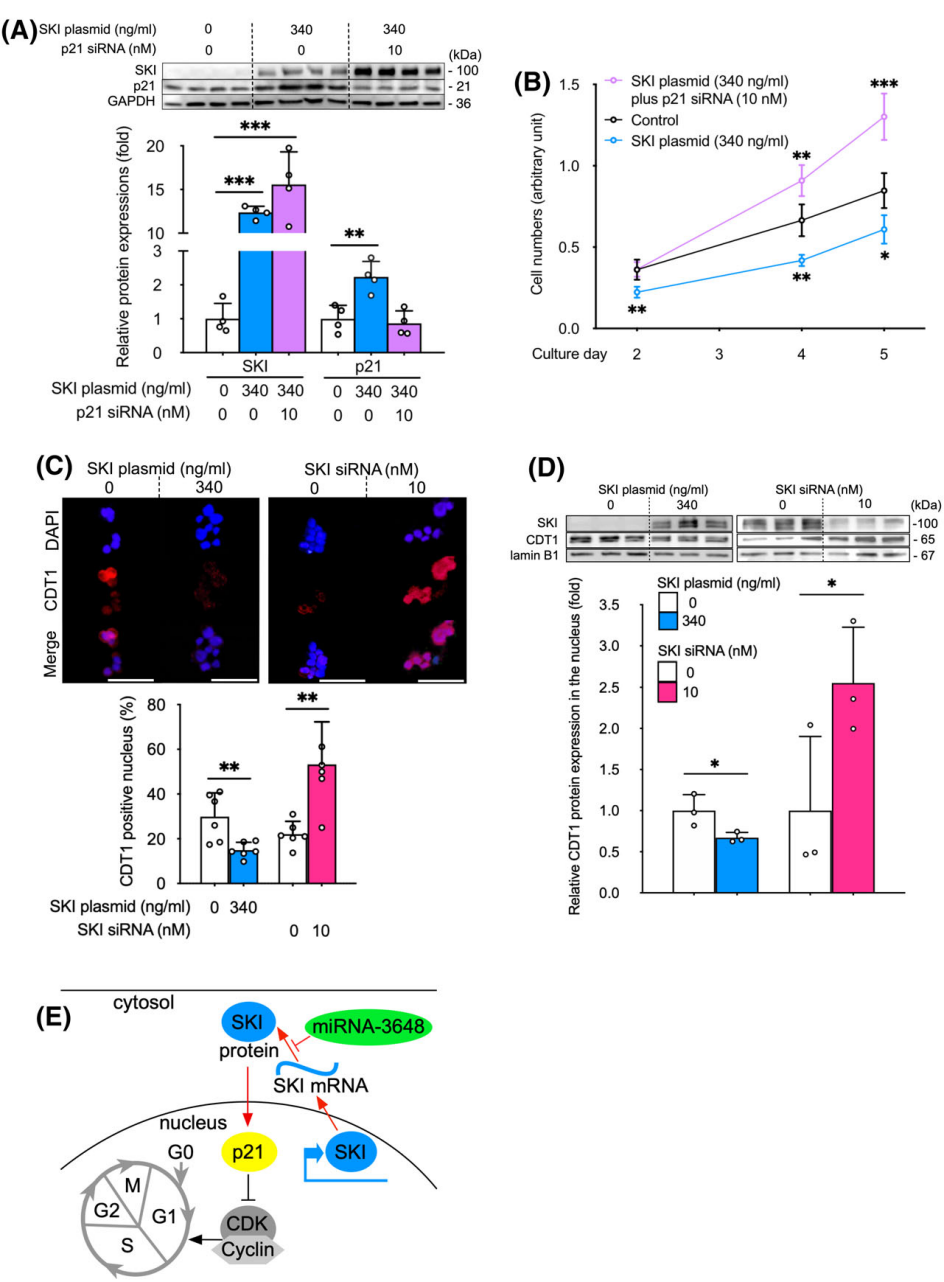
Sloan Kettering Institute suppresses cell proliferation via p21 upregulation and inhibits nuclear distribution of chromatin licensing and DNA replication factor 1 (CDT1) in human cholangiocarcinoma cells. (A) Western blot (upper) and quantitation (lower) of SKI and p21 protein expression (*n* = 4) and (B) cell proliferation assays using CCK‐8 in OZ cells after co‐transfection with 340 ng·mL^−1^ of SKI plasmid and 10 nm of p21 siRNA (*n* = 4). (C) Immunofluorescence analysis (upper) and quantitation (lower, *n* = 6) of CDT1 in SKI‐overexpressing (0 or 340 ng·mL^−1^ plasmid) and SKI‐knockdown (0 or 10 nm siRNA) OZ cells. Scale bar, 50 μm. (D) Western analysis (upper) and quantitation (lower) of CDT1 in nuclei of SKI‐overexpressing (0 or 340 ng·mL^−1^ plasmid) and SKI‐knockdown (0 or 10 nm siRNA) OZ cells (*n* = 3). (E) Schematic diagram of SKI–p21 signaling pathway in human cholangiocarcinoma cells. Red arrows show the findings of this study. GAPDH (A) or lamin B1 (D) served as loading controls. Symbols and bars represent the mean ± SD (A, C, D) or the mean ± standard error (B). All cells were transfected with equal amounts of total plasmid or RNA through the addition of empty plasmid or siCNT, respectively (A–D). *P < 0.05, **P < 0.01, ***P < 0.001; one‐way ANOVA with Dunnett's test (A, B) or unpaired *t*‐test (C, D).

To verify the involvement of p21 in CC cell proliferation, we performed p21 knockdown in SKI‐overexpressing OZ cells. The upregulation of p21 protein expression (Fig. 4A) and inhibition of cell proliferation on culture days 2–5 (Fig. 4B) observed in cells transfected with SKI plasmid (340 ng·mL^−1^) were not observed in cells co‐transfected with SKI plasmid and p21 siRNA (10 nm). These results suggest that p21 is involved in CC cell proliferation, which is suppressed by SKI–p21 signaling.

p21 is a CDK inhibitor and serves as a checkpoint that monitors each phase of the cell cycle, particularly G1 and G2, in cancer cells [37]. Our preliminary human CC flow cytometry analysis showed significant changes in fluorescence intensity during the S/G2/M phase according to SKI expression, including a significant decrease when SKI is overexpressed and a significant increase when SKI is knocked down (Fig. S4). CDT1, a DNA replication factor, is expressed at high levels during G1 but is degraded during S [38]. A decrease in the nuclear expression of CDT1 was observed in SKI‐overexpressing OZ cells compared with control cells (2.0‐fold decrease assessed by fluorescence immunostaining; Fig. 4C; 1.49‐fold decrease assessed by western analysis; Fig. 4D). SKI knockdown (10 nm siRNA) in OZ cells showed the opposite effects, including a 2.76‐fold increase in CDT1‐positive nuclear fluorescence immunostaining (Fig. 4C) and a 2.55‐fold increase in CDT1 protein expression, as assessed by western blot (Fig. 4D). p21‐mediated G1 arrest has been reported by *in vitro* non‐small cell lung cancer studies [39]. For the first time, our data indicate that SKI–p21 signaling underlies G1 arrest to suppress human intrahepatic CC growth (Fig. 4E).

p27 and ERK1/2 proteins are also involved in cell growth [12, 34]. In this study, SKI overexpression increased p27 protein levels but not *p27* mRNA levels. By contrast, SKI overexpression increased the mRNA levels but not the protein levels of the ERK1/2‐specific‐MAPK phosphatases *DUSP2* and *DUSP6*. Generally, mRNA levels are correlated with protein levels. However, in cancer cells, post‐transcriptional gene regulation has been shown to disrupt the correlation between mRNA and protein levels [40]. We also found that SKI‐overexpressing OZ cells tended to aggregate and could not be sorted smoothly for flow cytometry analyses, which may be due to the abundant production of mucin by this cell line [41, 42]. Therefore, we avoided the use of this cell for flow cytometric analyses and instead used the KKU100 cell line, as shown in the supplemental data. The discrepancies observed during the analyses of these CC cell lines do not alter the overall conclusions or quality of the study findings, but we hope to clarify these issues in future to better understand all aspects of CC.

In reverse translational research, in which clinical specimens and clinical information are collected from patients and analyzed to identify target genes, specimen quality is of utmost importance. To identify useful tumor suppressor genes, we first extracted the RNA from a wide range of clinical specimens, including patients younger than 50 years old, who are less affected by aging. Our study had some limitations; however, our data suggest that SKI–p21 signaling may act as a brake on cell cycle progression in this disease, suppressing cell proliferation. Although the functions mediated by this signaling pathway require validation in animal models, SKI may represent a new CC suppressor with the potential to effectively control CC progression. We are currently working to identify proteins that directly intervene in the SKI–p21 signaling pathway and to identify 8–10 bp DNA sequences that can serve as therapeutic RNA sponges, able to adsorb miRNAs that inhibit SKI and p21 functions. We hope that this signaling pathway will be recognized as a new therapeutic target candidate for intrahepatic CC.

## Conflict of interest

The authors declare no conflict of interest.

## Author contributions

EK and TM involved in conception and design; EK, SD, MK, HM, KK, RK, AH, HF, SU‐K, S Tanaka, S Takemura, AT, K Iwaisako, and SK involved in sample collection; EK, TM, AD, NO, HY, and HU involved in performance of the experiments; EK, TM, ME, YHT, K Ikeda, and NK involved in data analysis and interpretation; EK and TM involved in draft writing. All authors made final approval of the manuscript.

## Supporting information


**Fig. S1.** RNA selection using microarray and computational analysis.

**Fig. S2.** Principal component (PC) analysis of tissue microRNA (miRNA) and mRNA datasets discriminates between hepatobiliary tumor types.

**Fig. S3.** Box plots for microRNAs selected by principal component analysis.

**Fig. S4.** Preliminary flow cytometry analysis of Sloan Kettering Institute gene–overexpressing human cholangiocarcinoma cells indicates a decrease in fluorescence intensity during the S/G2/M phases.

**Table S1.** Sample information.

**Table S2.** 24 human microRNAs with differential expression among hepatobiliary tumors and their target mRNAs.

**Table S3.** 141 human mRNAs with differential expression among hepatobiliary tumors selected by principal component analysis.

**Table S4.** Cancer‐related pathways associated with microRNAs selected by principal component analysis.

**Table S5.** Cancer‐related pathways associated with mRNAs selected by target database.

**Table S6.** Cancer‐related pathways associated with mRNAs selected by principal component analysis.

## Data Availability

All microarray data were deposited in NCBI's Gene Expression Omnibus https://www.ncbi.nlm.nih.gov/geo/ and are accessible through GEO Series accession number GSE209875.
