# Supplementary material for: Suppression of intrahepatic cholangiocarcinoma cell growth by SKI via upregulation of the CDK inhibitor p21
Source: FEBS Open Bio. 2022 Sep 26;12(12):2122–35. doi: 10.1002/2211-5463.13489 (PMC9714377; doi:10.1002/2211-5463.13489)
Supplement: Supplementary file 1 — Fig. S1. RNA selection using microarray and computational analysis. [file FEB4-12-2122-s006.pdf]

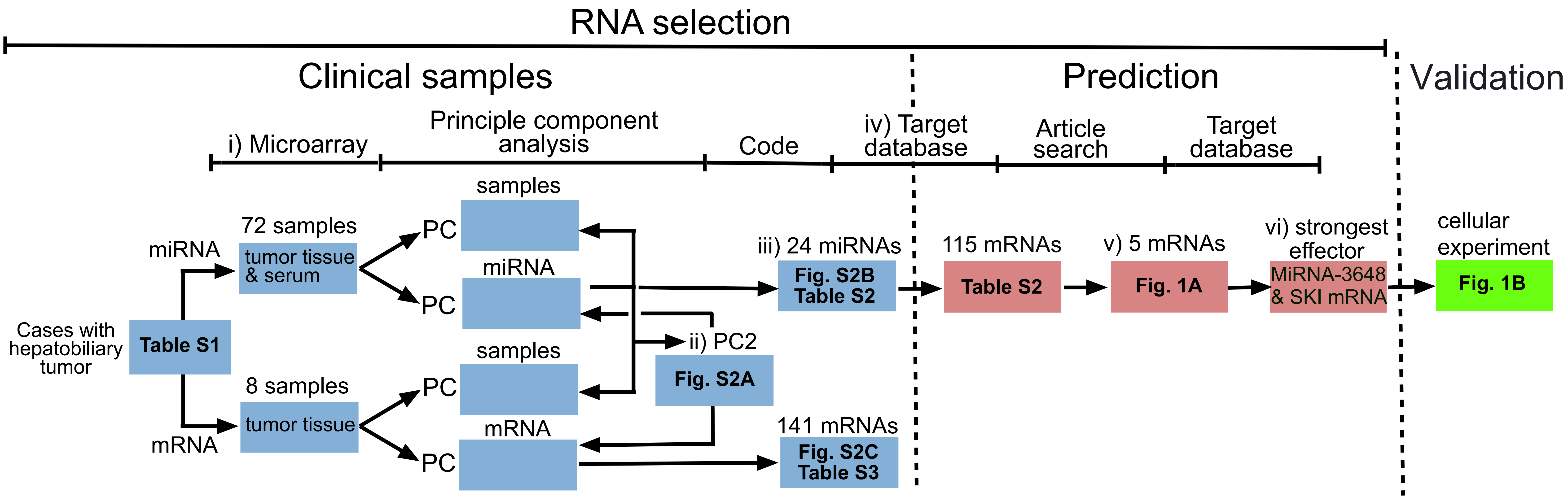

Fig. S1. RNA selection using microarray and computational analysis. (i) Raw data released to GEO. (ii) PC2 was selected because its microRNA (miRNA)–mRNA negative correlation was higher than that of PC1. (iii) MiRNAs selected by released R code CCA/sample.R at main ・ tagtag/CCA. (iv) Selection of mRNAs that satisfy the two database scores [context+ score ≤ −0.4 (TargetScan ver. 7) and target score ≥ 60 (miRDB ver. 5)]. (v) mRNAs selected by citations associated with non-cholangiocarcinoma digestive cancers. (vi) The pair showing the lowest context+ score out of 5 pairs. PC, Principle component.
