# Supplementary material for: Suppression of intrahepatic cholangiocarcinoma cell growth by SKI via upregulation of the CDK inhibitor p21
Source: FEBS Open Bio. 2022 Sep 26;12(12):2122–35. doi: 10.1002/2211-5463.13489 (PMC9714377; doi:10.1002/2211-5463.13489)
Supplement: Supplementary file 2 — Fig. S2. Principal component (PC) analysis of tissue microRNA (miRNA) and mRNA datasets discriminates between hepatobiliary tumor types. [file FEB4-12-2122-s004.pdf]

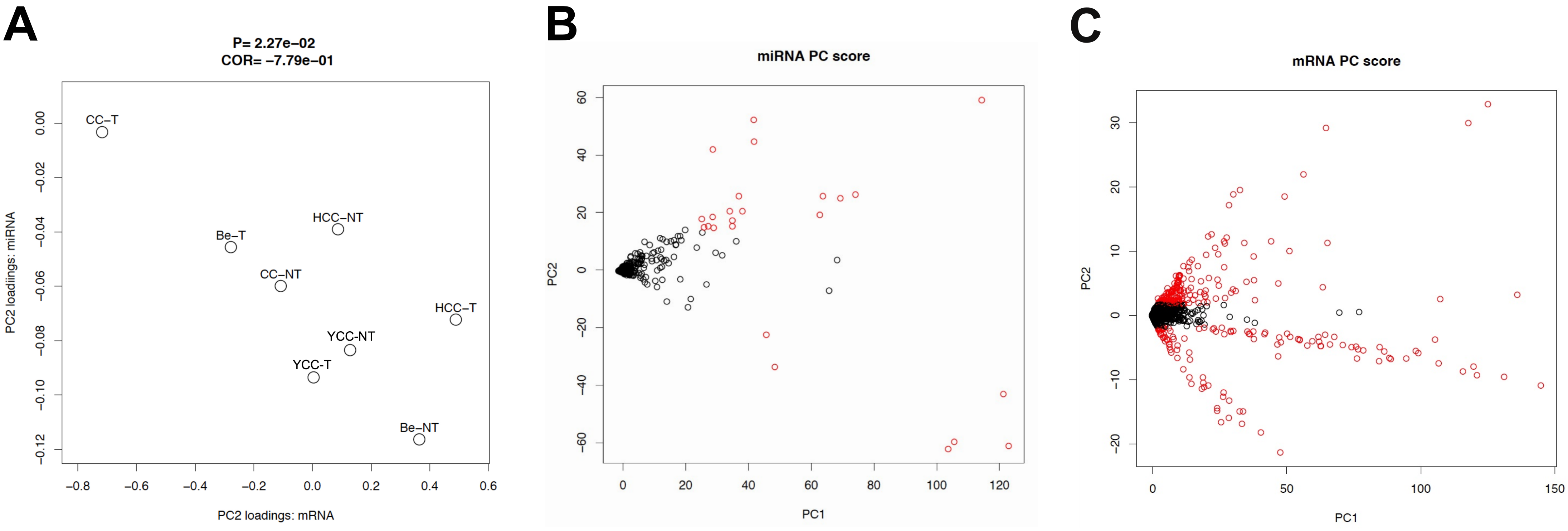

Fig. S2. Principal component (PC) analysis of tissue microRNA (miRNA) and mRNA datasets discriminates between hepatobiliary tumor types. (A) The axes of two PC2s with a negative correlation between the expression patterns of mRNAs and miRNAs in a two-dimensional model. CC, cholangiocarcinoma; YCC, cholangiocarcinoma of young individuals (younger than 50 years old); HCC, hepatocellular carcinoma; Be, benign hepatobiliary tumor; T, tumor; NT, non-tumor; (B) Red circles indicate significant miRNAs (n = 24) and (C) mRNAs (n = 141), using an adjusted $P < 0.01$, according to comparisons among tumor groups in a two-dimensional PC plot.
