# Supplementary material for: Suppression of intrahepatic cholangiocarcinoma cell growth by SKI via upregulation of the CDK inhibitor p21
Source: FEBS Open Bio. 2022 Sep 26;12(12):2122–35. doi: 10.1002/2211-5463.13489 (PMC9714377; doi:10.1002/2211-5463.13489)
Supplement: Supplementary file 3 — Fig. S3. Box plots for microRNAs selected by principal component analysis. [file FEB4-12-2122-s008.pdf]

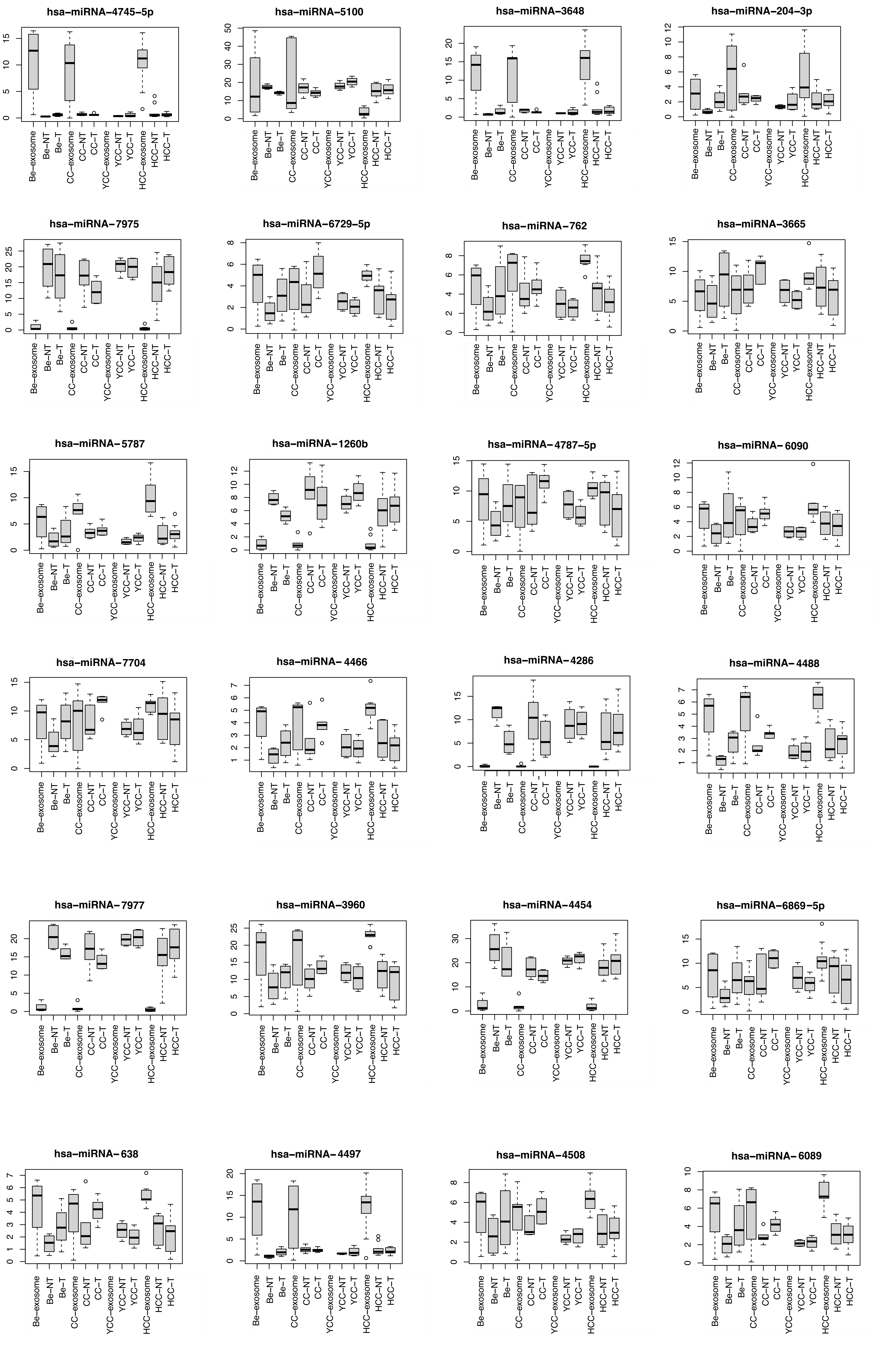

Fig. S3. Box plots for microRNAs (miRNAs) selected by principal component analysis. Expression patterns for the 24 miRNAs used to discriminate between hepatobiliary tumor and non-tumor samples. Vertical left axis shows miRNA expression levels. Adjusted *P*-values are indicated in Table S2. hsa, homo sapiens (human); Be, benign hepatobiliary tumor; CC, cholangiocarcinoma; YCC, cholangiocarcinoma of young individuals (younger than 50 years old); HCC, hepatocellular carcinoma; T, tumor; NT, non-tumor.
