# Supplementary material for: Suppression of intrahepatic cholangiocarcinoma cell growth by SKI via upregulation of the CDK inhibitor p21
Source: FEBS Open Bio. 2022 Sep 26;12(12):2122–35. doi: 10.1002/2211-5463.13489 (PMC9714377; doi:10.1002/2211-5463.13489)
Supplement: Supplementary file 4 — Fig. S4. Preliminary flow cytometry analysis of Sloan Kettering Institute gene–overexpressing human cholangiocarcinoma cells indicates a decrease in fluorescence intensity during the S/G2/M phases. [file FEB4-12-2122-s003.pdf]

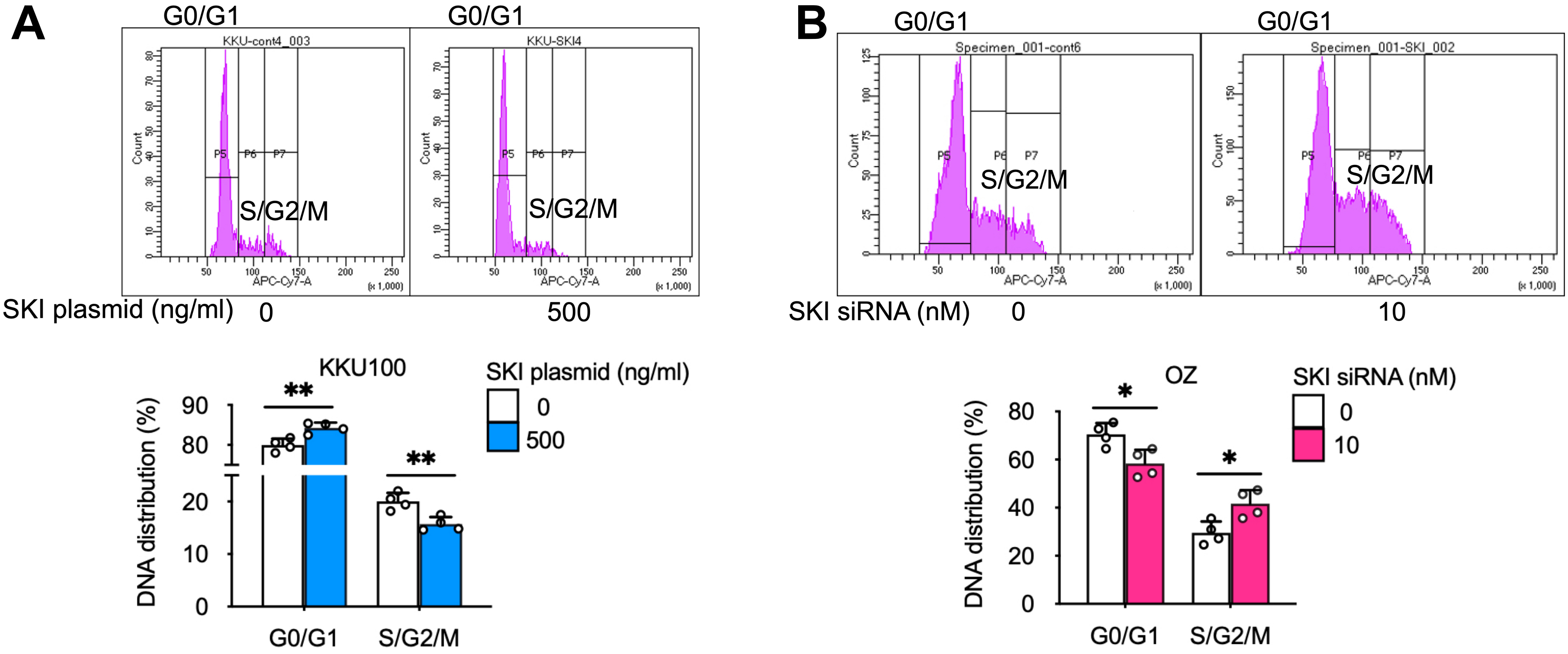

Fig. S4. Preliminary flow cytometry analysis of Sloan Kettering Institute (SKI)-overexpressing human cholangiocarcinoma cells indicates a decrease in fluorescence intensity during the S/G2/M phases. Flow cytometric analysis (upper) and quantitation (lower) of APC fluorescence intensity in the SKI-overexpressing (500 ng/ml plasmid) KKU100 cells **(A,** 1.27-fold decrease in distribution rates during S/G2/M phases**)** or SKI-knockdown (10 nM siRNA) OZ cells **(B,** 1.41-fold increase in distribution rates during S/G2/M phases**)** and control cells. Quantitation by cell cycle DNA distribution: P5 for G0/G1; P6 plus P7 for S/G2/M (n=4). All cells were transfected with equal amounts of total plasmid or RNA through the addition of empty plasmid or siCNT, respectively. Symbols and bars represent the mean ± SD. *p <0.05, **p <0.01; unpaired t-test. Abbreviation: APC-Cy7 A, allophycocyanin and a cyanine dye Cy7 A.
