# Supplementary material for: Suppression of intrahepatic cholangiocarcinoma cell growth by SKI via upregulation of the CDK inhibitor p21
Source: FEBS Open Bio. 2022 Sep 26;12(12):2122–35. doi: 10.1002/2211-5463.13489 (PMC9714377; doi:10.1002/2211-5463.13489)
Supplement: Supplementary file 8 — Table S4. Cancer‐related pathways associated with microRNAs selected by principal component analysis. [file FEB4-12-2122-s010.docx]

| **TABLE S4. Cancer-related pathways associated with microRNAs selected by principal component analysis** | | | | |
| --- | --- | --- | --- | --- |
| Pathway name | Source | *P*-value | *No. of genes | *No. of microRNAs |
| Colorectal cancer | KEGG | 5.45E−05 | 12 | 8 |
| Viral carcinogenesis | KEGG | 1.94E−02 | 22 | 6 |
| Endometrial cancer | KEGG | 2.04E−02 | 8 | 7 |
| Chronic myeloid leukemia | KEGG | 4.25E−02 | 11 | 4 |
| *Genes and microRNAs related to each pathway predicted by mirPath.  KEGG, Kyoto Encyclopedia of Genes and Genomes | | | | |
