# Supplementary material for: Suppression of intrahepatic cholangiocarcinoma cell growth by SKI via upregulation of the CDK inhibitor p21
Source: FEBS Open Bio. 2022 Sep 26;12(12):2122–35. doi: 10.1002/2211-5463.13489 (PMC9714377; doi:10.1002/2211-5463.13489)
Supplement: Supplementary file 9 — Table S5. Cancer‐related pathways associated with mRNAs selected by target database. [file FEB4-12-2122-s001.docx]

| **TABLE S5. Cancer-related pathways associated with mRNAs selected by target database** | | | | |
| --- | --- | --- | --- | --- |
| Pathway name | Source | *P*-value | Overlapping genes | *No. of genes |
| Hepatitis C and Hepatocellular Carcinoma | Wikipathways | 1.98E−02 | NM_001130713; NM_000610; NM_001001389; NM_001001390; NM_001001391; NM_001001392;  [NM_001202555](https://www.ncbi.nlm.nih.gov/nuccore/NM_001202556.2); [NM_001202556](https://www.ncbi.nlm.nih.gov/nuccore/NM_001202556.2);  [NM_001202557](https://www.ncbi.nlm.nih.gov/nuccore/NM_001202556.2) | 56 |
| Endometrial cancer | KEGG | 2.14E−02 | [NM_001351273](https://www.ncbi.nlm.nih.gov/nuccore/NM_001351273.1) | 58 |
| Endometrial cancer | Wikipathways | 2.32E−02 | [NM_001351273](https://www.ncbi.nlm.nih.gov/nuccore/NM_001351273.1) | 63 |
| Basal cell carcinoma | KEGG | 2.32E−02 | [NM_001351273](https://www.ncbi.nlm.nih.gov/nuccore/NM_001351273.1) | 63 |
| Chromosomal and microsatellite instability in colorectal cancer | Wikipathways | 2.69E−02 | [NM_001351273](https://www.ncbi.nlm.nih.gov/nuccore/NM_001351273.1) | 73 |
| Colorectal cancer | KEGG | 3.16E−02 | [NM_001351273](https://www.ncbi.nlm.nih.gov/nuccore/NM_001351273.1) | 86 |
| LncRNA involvement in canonical Wnt signaling and colorectal cancer | Wikipathways | 3.56E−02 | [NM_001351273](https://www.ncbi.nlm.nih.gov/nuccore/NM_001351273.1) | 97 |
| *Genes related to each pathway predicted by Integrated Molecular Pathway Level Analysis.  KEGG, Kyoto Encyclopedia of Genes and Genomes; lncRNA, long non-coding RNA | | | | |
