# Supplementary material for: Suppression of intrahepatic cholangiocarcinoma cell growth by SKI via upregulation of the CDK inhibitor p21
Source: FEBS Open Bio. 2022 Sep 26;12(12):2122–35. doi: 10.1002/2211-5463.13489 (PMC9714377; doi:10.1002/2211-5463.13489)
Supplement: Supplementary file 10 — Table S6. Cancer‐related pathways associated with mRNAs selected by principal component analysis. [file FEB4-12-2122-s009.docx]

| **TABLE S6. Cancer-related pathway associated with mRNAs selected by principal component analysis** | | | | |
| --- | --- | --- | --- | --- |
| Pathway name | Source | *P*-value | Overlapping genes | *No. of genes |
| Chemical carcinogenesis | KEGG | 3.51E−02 | NM_001075; NM_000669; NM_000670 | 83 |
| *Genes related to the pathway predicted by Integrated Molecular Pathway Level Analysis. KEGG, Kyoto Encyclopedia of Genes and Genomes | | | | |
